# A Role for Allantoate Amidohydrolase (AtAAH) in the Germination of *Arabidopsis thaliana* Seeds

**DOI:** 10.1093/pcp/pcac103

**Published:** 2022-07-21

**Authors:** Farzaneh Yazdanpanah, Leo A J Willems, Hanzi He, Henk W M Hilhorst, Leónie Bentsink

**Affiliations:** Wageningen Seed Science Centre, Laboratory of Plant Physiology, Wageningen University, Wageningen 6708 PB, The Netherlands; Wageningen Seed Science Centre, Laboratory of Plant Physiology, Wageningen University, Wageningen 6708 PB, The Netherlands; Wageningen Seed Science Centre, Laboratory of Plant Physiology, Wageningen University, Wageningen 6708 PB, The Netherlands; Wageningen Seed Science Centre, Laboratory of Plant Physiology, Wageningen University, Wageningen 6708 PB, The Netherlands; Wageningen Seed Science Centre, Laboratory of Plant Physiology, Wageningen University, Wageningen 6708 PB, The Netherlands

**Keywords:** Ammonia, *Arabidopsis thaliana*, Block of germination, Nitrogen, Purine pathway, Seed dormancy

## Abstract

Seed dormancy is a very complex trait controlled by interactions between genetic and environmental factors. Nitrate is inversely correlated with seed dormancy in Arabidopsis. This is explained by the fact that seed dry storage (after-ripening) reduces the need for nitrogen for germination. When nitrate is absorbed by plants, it is first reduced to nitrite and then to ammonium for incorporation into amino acids, nucleic acids and chlorophyll. Previously, we showed that *ALLANTOATE AMIDOHYDROLASE* (*AtAAH*) transcripts are up-regulated in imbibed dormant seeds compared with after-ripened seeds. AAH is an enzyme in the uric acid catabolic pathway which catalyzes the hydrolysis of allantoate to yield CO_2_, NH_3_ and *S*-ureidoglycine. This pathway is the final stage of purine catabolism, and functions in plants and some bacteria to provide nitrogen, particularly when other nitrogen sources are depleted. *Ataah* mutant seeds are more dormant and accumulate high levels of allantoate, allantoin and urea, whereas energy-related metabolites and several amino acids are lower upon seed imbibition in comparison with Columbia-0. *AtAAH* expression could be detected during the early stages of seed development, with a transient increase around 8 d after pollination. *AtAAH* expression is the highest in mature pollen. The application of exogenous potassium nitrate can partly complement the higher dormancy phenotype of the *Ataah* mutant seeds, whereas other nitrogen sources cannot. Our results indicate that potassium nitrate does not specifically overcome the alleviated dormancy levels in *Ataah* mutant seeds, but promotes germination in general. Possible pathways by which AtAAH affects seed germination are discussed.

## Introduction

Seed dormancy is an adaptive trait that regulates the timing of seed germination. Environmental factors, including light (i.e. light quality and photoperiod), temperature, water, nutrients, the duration of seed storage as well as growth conditions of the mother plant, influence the level of seed dormancy (Bewley 1997, Bewley et al. 2013). Nitrogen, which is essential for plant growth, development and reproduction, is inversely correlated with seed dormancy in Arabidopsis. Conditions that favor nitrate accumulation in mother plants and seeds lead to lower seed dormancy levels. Alboresi et al. (2005) showed that this effect is mediated by nitrate signaling and not the nutritional contribution of nitrate. Nitrate accumulation in seeds reduces the gibberellic acid requirement for germination and accelerates the decrease in ABA levels during early seed imbibition (Alboresi et al. 2005). It was shown that nitrate may act on seed dormancy, at least in part, via the production of nitric oxide (NO) and its subsequent effects on levels of ABA in seeds (Arc et al. 2013).

Seed dry storage, also referred to as after-ripening, leads to a reduction of seed dormancy. After-ripening can also be considered as a widening of the germination window, allowing seeds to germinate in conditions that inhibit the germination of freshly harvested seeds (Finch-Savage and Leubner-Metzger 2006, Soppe and Bentsink 2020). As a result, after-ripening also reduces the nitrogen requirement for germination (Finch‐Savage et al. 2007).

**Fig. 1:**
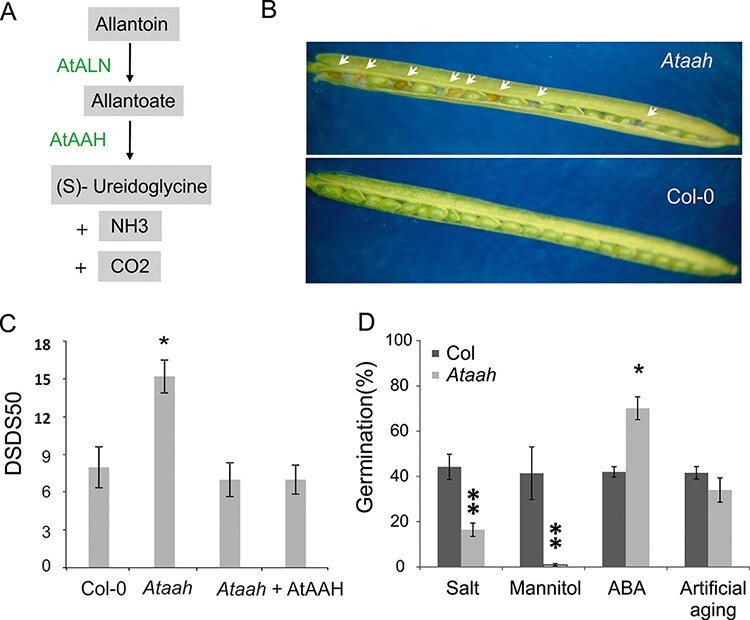
The *S*-allantoin degradation pathway and the effect of a defective AtAAH enzyme on Arabidopsis seed phenotypes. (A) Schematic presentation of *S*-allantoin degradation in plants. Conversions in the pathway and the enzymes responsible are indicated. (B) Open silique of the *Ataah* mutant in comparison with Columbia-0 (Col-0). The mutant contains several defective seeds indicated by the arrows. (C) Seed dormancy levels expressed as days of seed dry storage to reach 50% of germination (DSDS50) of *Ataah*, and of Col-0 and two independent *Ataah* complementation lines. (D) Seed performance of after-ripened seeds (germination after artificial aging and germination in stress conditions including: salt 130 mM; mannitol (−1 MPa) and ABA (0.15 µM)). Shown are averages of four biological replicates and their SE. Significant differences between *Ataah* and Col-0 are indicated (*P < 0.05 and **P < 0.01).

Plants can take up nitrogen from the soil. Generally, they take it up in the form of ammonium or amino acids, but nitrate is preferred by plants that are adapted to higher pH and more aerobic soils (for a review, see Maathuis 2009). After absorbance, nitrate is usually first reduced to nitrite and then to ammonium before it is incorporated into amino acids, nucleic acids and chlorophyll (Smil 2000). Moreover, the nitrogen that is incorporated in pyrimidine and purine bases in the DNA and RNA of senescing leaves can be remobilized, and as such serve as a nitrogen source for the production of leaves and seeds (Malagoli et al. 2005, Diaz et al. 2008). The metabolic pathways required for this remobilization is largely conserved between plants, fungi and bacteria, involves a series of enzymatic steps and takes place in the cytosol, peroxisome and the endoplasmic reticulum (Vogels and Van der Drift 1976, Tipton 2006). Purine catabolism leads to allantoin and allantoate, compounds that have a favorable N:C ratio to serve nitrogen transport and storage. In plants, the first step of the ureide (allantoin and allantoate) metabolic pathway occurs in the cytoplasm and leads to xanthine. The next step, which is the oxidation of xanthine to uric acid, occurs in the cytoplasm. After this, uric acid is transported into the peroxisome and converted into *S*-allantoin by three enzymatic steps (Ramazzina 2006). The hydrolysis of *S*-allantoin by ALLANTOINASE (ALN) to allantoate and the breakdown of allantoate by ALLANTOATE AMIDOHYDROLASE (AAH) to usable nitrogen occur in the endoplasmic reticulum (Hanks et al. 1981) (**Fig. 1A**). Arabidopsis plants defective in AAH prevent the remobilization of purine nitrogen and are not able to grow when only allantoin is provided as the nitrogen source (Werner et al. 2008).

Possible involvement of the allantoate pathway in seed dormancy was suggested from studies on near isogenic lines that contain *DELAY OF GERMINATION* loci (*DOG* NILs). Seeds of these lines which displayed increased dormancy levels, induced by growing plants in low nitrate and low temperature conditions, have reduced seed allantoin and urea contents when compared with seeds developed in control conditions (He et al. 2016). Transcriptome analyses on the same seeds showed an up-regulation of ALN gene expression (He et al., 2016), which is probably a feedback reaction caused by the lack of ammonia. Moreover, it is known that ABA is required for the induction of seed dormancy (Hilhorst 1995) and that there is a link between purine catabolism and ABA. Studies using Arabidopsis mutants defective in purine catabolism have shown that the intermediary metabolite allantoin stimulated ABA production and enhanced abiotic stress tolerance (Watanabe et al. 2014a, Watanabe et al. 2014b). Furthermore, *Ataln* mutant seeds displayed deep dormancy relative to wild-type seeds, identifying AtALN as a negative regulator of seed dormancy (Piskurewicz et al. 2016).

We have identified *AtAAH* transcripts to be up-regulated in imbibed dormant seeds (Yazdanpanah et al. 2017). Here we investigated whether AtAAH plays a role in seed dormancy. Interestingly, seeds of the Arabidopsis loss-of-function *Ataah* mutant showed increased seed dormancy compared with wild-type Columbia-0 (Col-0). This dormancy could be partly compensated by the application of exogenous potassium nitrate, either during the growth of the mother plant or during imbibition of the seeds.

## Results

### Loss of function of *AtAAH* leads to partially defective seed maturation and increased seed dormancy

Homozygous T-DNA knock-out plants of *AtAAH* (At4G20070) grow normally. However, lack of functional *AtAAH* leads to a certain amount of seed abortion (**Fig. 1B**). A possible role for *AtAAH* during seed development is underlined by its high expression in pollen (http://bar.utoronto.ca/efp/cgi-bin/efpWeb.cgi) (**Supplementary Fig. S1**). *Ataah* mutant seeds display increased dormancy, expressed as a higher after-ripening requirement (DSDS50) and a higher sensitivity to salt (NaCl) and mannitol. The mutation did not affect seed longevity (germination after artificial aging) but the seeds were more tolerant to ABA (**Fig. 1D**). The similar pattern between salt and mannitol indicates that the inhibition of germination is likely to be an osmotic effect. The dormancy phenotype of the mutant is complemented by the introduction of wild-type AtAAH under a 35S promoter into the *Ataah* mutant background (**Fig. 1C**). This confirmed that the observed phenotypes in the mutant were caused by disruption of the *AAH* gene and are not due to an additional T-DNA insert or spontaneous mutation (O’Malley and Ecker 2010, Gase et al. 2011).

### Expression of *AtAAH* during plant development

**Fig. 2:**
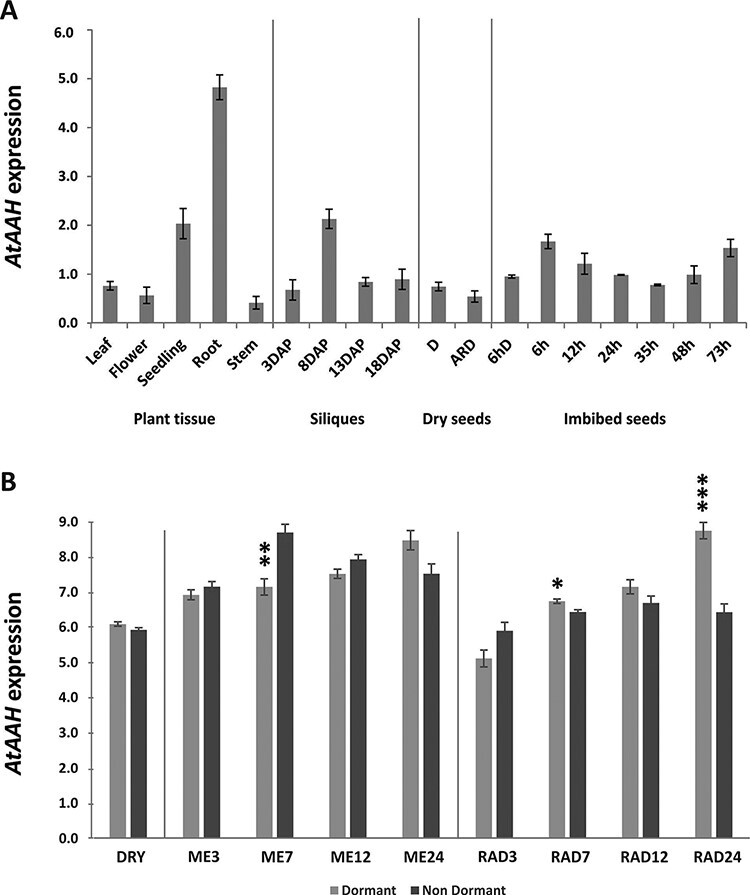
Expression pattern of *ALLANTONATE AMIDOHYDROLASE* (*AtAAH*) during plant development. (A) Mean relative expression levels of *AtAAH* measured by RT–qPCR in tissues of the Columbia accession leaf (L), flower (F), seedling (SE), root (R) and stem (ST) in siliques 3, 8 13 and18 d after pollination (DAP), in dormant dry (D) and 6 h imbibed seeds (6hD), and across the germination time course in after-ripened dry (ARD) seeds and 6, 12, 24, 35, 48 and 73 h after imbibition. Expression values are normalized by the expression of two reference genes that are stably expressed in dry seeds: At4g12590 and At4g34270. (B) *AtAAH* expression patterns measured by qbasePLUS software in dormant and non-dormant (after-ripened) dry Cvi seeds and the micropylar and chalazal endosperm (ME) and radicle and hypocotyl (RAD) at 3, 7, 12 and 24 h after imbibition. Asterisks indicate significant differences between dormant and non-dormant in *AtAAH* expression (***P < 0.001; **P < 0.01; *P < 0.05).

The expression pattern of *AtAAH* during plant development was analyzed by reverse transcription–quantitative PCR (RT–qPCR) in Col-0. In addition to its expression in pollen, the gene is expressed throughout plant development, but it is especially high in seedlings, roots and in siliques, 8 d after pollination (**Fig. 2A**).

Earlier, we identified *AtAAH* as a dormancy-up gene based on the higher expression in dormant compared with after-ripened seeds (Yazdanpanah et al. 2017). To investigate how the higher expression in dormant vs. after-ripened seeds matches the more dormant phenotype of the knock-out mutant, the expression pattern of *AtAAH* was analyzed in Cvi (Cape Verde Islands) seeds. This analysis shows that *AtAAH* expression in dry seeds is almost similar for dormant and non-dormant seeds, but it increased upon imbibition in both the micropylar and chalazal endosperm (ME), as well as the radicle and hypocotyl (RAD) of dormant seeds, but significantly less in non-dormant seeds (**Fig. 2B**).

### Exogenous potassium nitrate partly rescues the *Ataah* phenotype

**Fig. 3:**
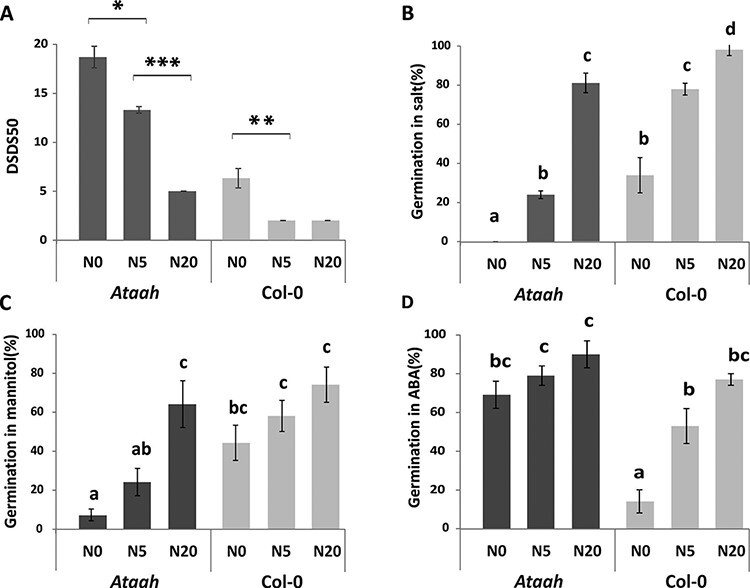
Effect of different nitrate regimes during seed maturation on seed performance. Seed performance of both *Ataah* and Col-0 is presented. (A) Dormancy level (DSDS50), (B) germination in salt (130 mM NaCl), (C) germination in mannitol (−1 MPa), (D) germination in ABA (0.15 μM) of seeds matured on plants exposed to different nitrate levels (N0, N5 and N20). Shown are averages of four biological replicates and their SE. Significant differences between *Ataah* and Col-0 are indicated by *** (*P* < 0.001) and different letters (Student *t*-tests; *P* < 0.05).

To find out whether the increased seed dormancy of the mutant is a direct result of the lack of available nitrate, we applied exogenous potassium nitrate (KNO_3_) to the mother plant during seed development and to the seeds during seed imbibition. Nitrate assimilation of parent plants during seed maturation affects the accumulation of nitrate in seeds. Different KNO_3_ regimes (N0, N5 and N20) resulted in corresponding (low in N0 and high in N20) nitrate levels in the mature seeds; these levels were similar for both the *Ataah* mutant and the wild type (**Supplementary Fig. S2A**). Treatment of the *Ataah* mutant with the highest nitrate level partly overcame the increased dormancy of the mutant compared with that of the wild-type Col-0 (**Fig. 3A**), but it could not prevent the seed abortion in the mutant. Similar results were obtained for the germination in salt and mannitol (**Fig. 3B**, **C**). The application of the highest nitrate concentration (20 mM) did completely abolish the difference between the wild type and mutant when germinated in mannitol (**Fig. 3C**). The germination of the mutant in ABA was not affected by the application of nitrate (**Fig. 3D**). Also the seed ABA levels did not differ either among the seeds that were grown in the different nitrate regimes or by mutant complementation (**Supplementary Fig. S2B and C**).

**Fig. 4:**
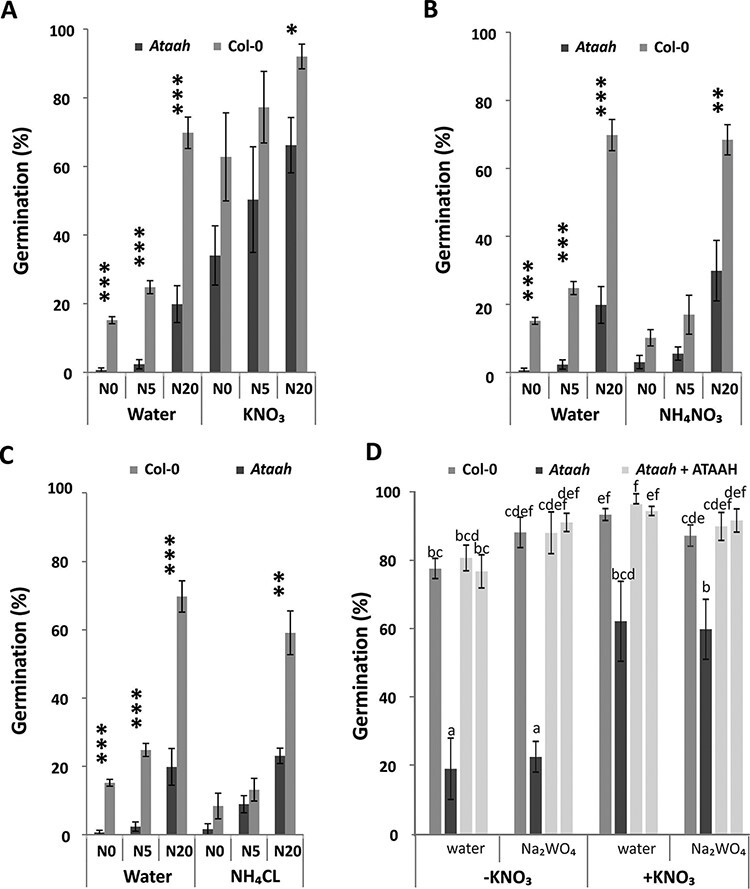
Effect of applying nitrogen-containing compounds during seed imbibition. Germination percentage of *Ataah* and Col-0 after applying (A) KNO_3_ (10 mM), (B) NH_4_NO_3_ (10 mM) and (C) NH_4_Cl (10 mM) during seed imbibition. Means of maximum germination percentage from four replicates are presented. Significant differences between *Ataah* and the control Col-0 are indicated (*P < 0.05, **P < 0.01 and ***P < 0.001). The experiments presented in (A–C) were all performed at the same time; the KNO_3_, NH_4_NO_3_ and NH_4_Cl treatments are compared with the same water control. (D) Germination percentage of Col-0, *Ataah* and two independent *Ataah* complementation lines in the presence of the nitrate reductase inhibitor tungsten (Na_2_WO_4_), with and without addition of 10 mM KNO_3_. The experiment was performed 28 d after seed harvest when the *Ataah* seeds had not yet after-ripened. Means of maximum germination percentage from four replicates are presented. Differences are indicated by different letters (Student *t*-tests; *P* < 0.05).

Freshly harvested (5 d after harvest) dry seeds that had developed under the three nitrate regimes were imbibed in three different nitrogen sources (nitrate or ammonium). For all genotypes, germination of seeds treated with KNO_3_ was higher (*P* < 0.05) as compared with the germination in water (**Fig. 4A**). Thus, both wild-type and mutant seeds responded to exogenous KNO_3_, of which the mutant response was more obvious. The application of KNO_3_ rescued the germination of the mutant, since the germination of the mutant with the addition of KNO_3_ is higher than that of the wild type in water for all three nitrogen seed maturation regimes. Imbibition on NH_4_NO_3_ and NH_4_Cl did not affect the germination percentage (**Fig. 4B**, **C**), although NH_4_ possibly had a toxic effect considering the fact that the non-germinating seeds turned dark (data not shown). Further it was investigated whether the effect of KNO_3_ is the result of nitrate signaling or of nitrate assimilation. For nitrate assimilation, the conversion of NO_3_ to NO_2_ by nitrate reductase (NR) is required. Inhibiting NR by tungstate (Hilhorst and Karssen 1989) during seed germination did not inhibit the effect of KNO_3_ on the partial complementation of the dormancy phenotype and therefore excludes an effect of nitrate as the nitrogen resource (**Fig. 4D**).

### Expression analysis of other genes in the purine pathway

**Fig. 5:**
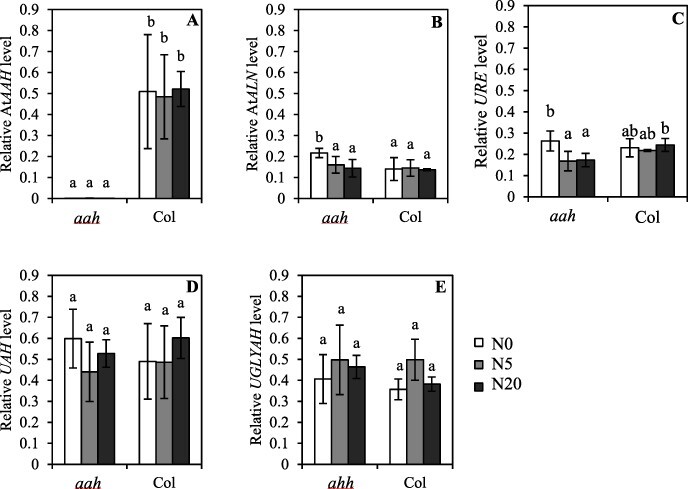
Relative expression of genes in the purine pathway related to allantoate degradation: *AtAAH* (A), *AtALN* (B), *URE* (C), *UAH* (D) and *UGLYAH* (E). Plant material consisted of seeds of Col and the *ataah* mutant matured on plants exposed to different nitrate levels. Means of relative expression level (reference genes At3g25800 and At4g34270) from four replicates are presented. Error bars represent the SEs, and different letters indicate statistically significant differences (Student *t*-tests; *P* < 0.05).

To investigate whether the loss of function of *ATAAH* affected other genes in the purine pathway (*AtALN*, allantoinase; *URE*, urea hydrolase; *UAH*, ureidoglycolate amidohydrolase; and *UGLYAH* ureidoglycine aminohydrolase), RT–qPCR was performed on seeds of the *Ataah* mutant and its wild type (Col-0) matured in the different nitrate regimes. These analyses confirmed the absence of the relevant mRNA in the *Ataah* T-DNA insertion mutant (**Fig. 5A**). Further, only *AtALN* and *URE* displayed slightly, but significantly, higher expression in seeds of the *Ataah* mutant which had developed under N0, as compared with the other nitrate regimes (**Fig. 5B**, **C**). *AtALN* expression in the mutant was also significantly higher at N0 than that in Col-0 at the same concentration (**Fig. 5B**).

### Metabolic changes in the *Ataah* loss-of-function mutant

**Fig. 6:**
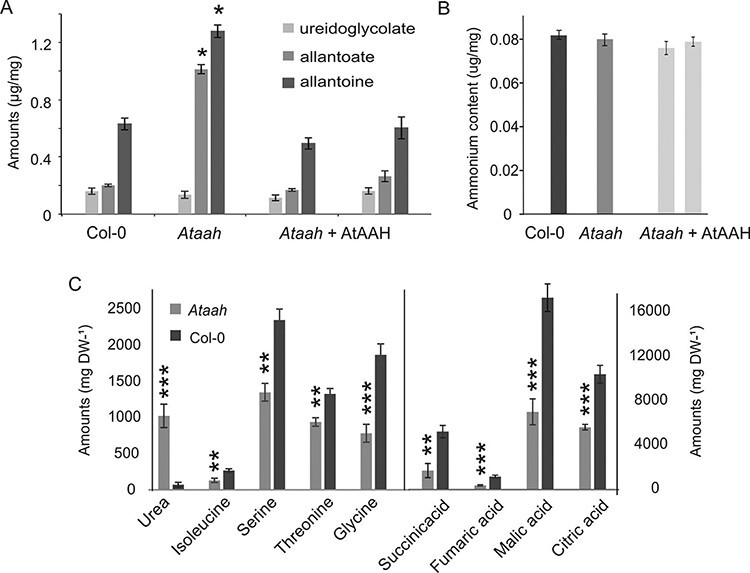
(A) Metabolic profiles of seeds of Col-0 and the *Ataah* mutant. Allantoate, allantoate and ureidoglycolate concentrations in Col-0, *Ataah* and two independent *Ataah* complementation lines. (B) Ammonium content of Col-0, *Ataah* and two independent *Ataah* complementation lines. (C) Metabolite levels (mg DW^−1^) in 24 h imbibed dormant seeds of Col-0 and the *Ataah* mutant. Asterisks indicate significant differences relative to the respective wild type (***P < 0.001; **P < 0.01).

The levels of ureidoglycolate, allantoin and allantoate were determined in dormant dry seeds of Col-0 wild type, the *Ataah* mutant and two independent *Ataah* complementation lines. This analysis showed that mutant seeds had significantly higher levels of allantoin and allantoate than the wild type (**Fig. 6A**). Moreover, complementation of the *Ataah* mutant with wild-type *AtAAH* reverted the mutant phenotype (**Fig. 6A**). There were no differences in ammonium contents between the Col-0 wild type, the *Ataah* mutant and two independent *Ataah* complementation lines (**Fig. 6B**).

GC-MS metabolite profiling of 24 h imbibed dormant seeds of Col-0 and the *Ataah* mutant revealed a higher urea abundance in the mutant (about 12 times more than Col-0). The contents of the amino acids serine, threonine, isoleucine and glycine were strongly reduced and there was a reduction in the organic acids malate, fumarate, citrate and succinate in the mutant as compared with the wild type (**Fig. 6C**).

## Discussion

The final steps of purine degradation have long been the focus of research, especially because in tropical legumes these reactions are central to nitrogen supply under nitrogen-fixing conditions. Among the enzymes involved, a key role for AtAAH for recycling purine ring nitrogen has been demonstrated (Werner et al. 2008). In the present study we identified increased seed dormancy in seeds of the *Ataah* mutant. This phenotype is similar to that of a knock-out mutant of another gene in the same pathway, ALLANTOINASE (AtALN) (Piskurewicz et al. 2016). Loss-of function of *AtALN* has been reported to activate ABA metabolism in response to drought and osmotic stress in *Ataln* mutant seedlings (Watanabe et al. 2014b). ABA measurements in freshly harvested *Ataah* and Col-0 seeds produced under different nitrate regimes and in the mutant and complementation lines revealed that changes in ABA levels are not the cause of the increased dormancy of the *Ataah* mutant seeds (**Supplementary Fig. S2BC**).

AtAAH was found as a dormancy-up gene (Yazdanpanah et al. 2017), which suggests that its expression in dormant seeds is induced to overcome the block of germination by increasing internal nitrogen resources. In agreement with this, Takagi and coauthors reported that *Ataln* and *Ataah* mutants are relatively inefficient at using N for carbon assimilation and dry matter production. Applying exogenous nitrate could restore the growth of the *Ataah* mutant but not of the *Ataln* mutants (Takagi et al. 2016). To investigate whether indeed lack of nitrogen caused the increased dormancy in *Ataah* seed, we applied nitrogen to either the growth or the germination medium (**Figs. 3** and **4**). Application of high nitrate (20 mM) to the growth solution rescued, although not completely, the dormancy phenotype of the mutant. The higher exogenous nitrate content also clearly promoted the germination of after-ripened *Ataah* seeds under salt and osmotic stress, but did not affect germination in the presence of ABA. *Ataah* mutant seeds were hypersensitive to ABA, which suggests that the phenotype of the mutant is not directly related to ABA and ABA sensitivity. Moreover, among the various nitrogen compounds applied during the imbibition of freshly harvested seeds, only KNO_3_ stimulated germination, and the highest concentration even completely rescued the dormancy phenotype of the mutant. In contrast to other species, ammonium nitrate did not promote germination in Arabidopsis seeds, but, on the contrary, probably exerted a toxic effect, as explained by the darkening of the seedlings. The fact that only nitrate affected the germination of the mutant and not the other nitrogen sources suggests an effect of nitrogen as a signaling molecule rather than a nutritional role. This is confirmed by the fact that there is no difference in germination percentage when nitrate reductase is inhibited by the application of tungstate (**Fig. 4D**). The dormancy-breaking effect of nitrogen as a signal is well known (Alboresi et al. 2005) and has been reported to be similar to that of other germination stimulators, such as after-ripening, light and stratification (Finch‐Savage et al. 2007). Apart from other signaling pathways, nitrate signaling may induce the expression of the ABA catabolic gene CYP707A2 (Ali-Rachedi et al. 2004, Alboresi et al. 2005, Matakiadis et al. 2009). Our investigations, as explained above, seem to exclude a role for ABA in the increased dormancy of the *Ataah* mutant.

Taken together, we hypothesize that the effect of KNO_3_ is explained by the general effect that nitrate has on promoting germination and thus does not specifically rescue the dormancy phenotype of the *Ataah* mutant. Factors that might explain the AtAAH germination phenotype are discussed below.

Wild-type plants exposed to low nitrogen conditions show reduced levels of allantoate, which supports the nutritional effect of nitrate and that ureides are a source of recycled nitrogen (Takagi et al. 2016). *Ataah* mutant seeds contain higher amounts of allantoin and allantoate due to the fact that these mutants lack a functional AtAAH which is required for the breakdown of these ureides (Todd and Polacco 2006). This is probably the reason for the lower abundance of the amino acids serine, threonine, isoleucine and glycine in *Ataah* seeds (**Fig. 3**). Consequently, the lack of these amino acids might inhibit the translation of transcripts that are necessary for germination. On the other hand, the reduced amino acid levels in the *Ataah* mutant seeds might be the result of, or the reason for, the higher sensitivity of the mutant seeds to salt and mannitol, since accumulation of compatible solutes such as amino acids is one of the mechanisms to deal with negative effects of salinity on plants (Giri 2011). Seeds of the *Ataah* mutant with increased dormancy also display a reduced energy metabolism and tricarboxylic acid (TCA) cycle activity, as compared with Col-0, and which can be concluded from the reduced amounts of malate, fumarate, citrate and succinate in the mutant compared with the wild type (**Fig. 6B**). It has been suggested that the TCA cycle is activated during seed germination (He et al. 2011). Thus, the low abundance of TCA cycle metabolites might be caused by the dormancy status of the mutant.

Moreover, there are several reports that consider allantoin and allantoate as reactive oxygen species (ROS) protectants (Takagi et al. 2016, Brychkova et al. 2008, Yesbergenova et al. 2005, Alamillo et al. 2010, Watanabe et al. 2014a, Irani and Todd 2016). These metabolites can act as scavengers of ROS and might contribute to stress protection during the maturation phase, dormancy and germination of seeds, as well as early growth of seedlings (Takagi et al. 2016). ROS have been shown to be important for overcoming seed dormancy (Oracz et al. 2007). Therefore, the overaccumulation of allantoin and allantoate in the *Ataah* mutant seeds may prevent the achievement of levels of ROS that are required to induce germination.

*Ataah* mutant seeds also accumulate urea, which might be explained by arginase activity. Arginine and ureides (such as allantoin and allantoate) represent the major potential sources of ammonium for incorporation into proteins as well as nucleic acids and a range of secondary metabolites (Trivedi 2006). Arginine breakdown is catalyzed by the mitochondrial arginase; this step involves arginine hydrolysis to ornithine and urea. In the *aah* mutant, allantoin and allantoate cannot be metabolized, the route via arginine might be activated and thus might result in urea accumulation.

In conclusion, the effect of AtAAH on seed germination is not completely revealed but the data presented here strongly suggest that AtAAH is required to support seed germination. When AtAAH is non-functional, this leads to an increase of seed dormancy. Preparing the seeds for seed germination already starts during seed maturation, which is also explained by the rather high expression of *AtAAH* during seed development and the up-regulation in dormant seeds (**Fig. 2A**). However, by far the highest transcript abundance was found in mature pollen (**Supplementary Fig. S1**). It is possible that the lack of this enzyme during early embryogenesis causes abortion of *Ataah* mutant seeds. Concerning the fact that siliques of mutated plants contain both normal and aborted seeds (**Fig. 1B**), it is more likely that abortion is a result of reduced fertilization efficiency of the pollen rather than being the consequence of a defect in embryogenesis of the mutant seeds.

## Materials and Methods

### Plant material

*Arabidopsis thaliana* wild accession Col-0 and the homozygous T-DNA insertion mutant *Ataah* (SALK_112631; At4g20070) were used for these analyses. The homozygous T-DNA insertion mutant was isolated by a PCR-based reverse genetic screen for T-DNA insertions in the corresponding gene (Alonso et al. 2003).

The complementation lines with homozygous transgene insertions, the *Ataah* mutant complemented with 35S-AtAAH-HAStrep, were described before by Werner et al. (2008).

### Plant growth conditions

Seeds were sown in Petri dishes on water-soaked filter paper followed by a 4 d cold treatment at 4°C, and transferred to a climate room at 22°C with continuous light for 3 d before planting. Germinated seedlings were grown on 4 × 4 cm Rockwool blocks in a growth chamber at 20°C/18°C (day/night) under a 16 h photoperiod of artificial light (150 μmol m^–2^ s^–1^) and 70% relative humidity. Plants were grown in a standard nutrient solution (**Supplementary Table S1**) and watered three times per week. For nitrate-treated plants, when flowering started, the KNO_3_ regime of plants was changed to 0, 5 and 20 mM, resulting in corresponding (low in N0, standard in N5 and high in N20) nitrate levels in the mature seeds, with four biological replicates containing four plants per replicate for each condition.

### Germination assays

Germination tests to monitor the release of seed dormancy were performed as described in Yazdanpanah et al. (2017) based on the germinator package developed by Joosen et al. (2010). In short, at several intervals during seed dry storage, until all seed batches had reached 100% germination, aliquots of 50–100 seeds of each genotype were evenly sown on a filter paper soaked with 0.7 ml of demineralized water in a 6 cm Petri dish. Petri dishes were placed in plastic trays containing a filter paper saturated with tap water and closed with transparent lids. Trays were incubated for 1 week in a climate chamber, illuminated by 38 W Philips TL84 fluorescent tubes at 8 W m^−2^ in continuous light at 22°C. After that, the total number and the number of germinated seeds was scored and percentages calculated.

Seed dormancy levels (DSDS50: days of seed dry storage required to reach 50% germination) were quantified as described in Yazdanpanah et al. (2019). In brief, germination tests were performed weekly until all seed batches had germinated to >90%. A generalized linear model with a logit link was adapted to calculate DSDS50 as previously introduced (He et al. 2014).

Germination under stress conditions was performed on fully after-ripened seeds as described in Yazdanpanah et al. (2019). Stress conditions were: osmotic stress (−1 MPa mannitol; Sigma-Aldrich), salt stress (130 mM NaCl; Sigma-Aldrich) and ABA stress (0.15 μM ABA; Duchefa Biochemie). ABA was dissolved in 10 mM MES buffer (Sigma-Aldrich) and the pH adjusted to 5.8. To determine seed longevity, an accelerated aging test was performed by incubating seeds above a saturated ZnSO_4_ solution (40°C, 85% relative humidity) in a closed tank for 5 d (ISTA, 2022). After incubation, the seeds were taken out and germinated on demineralized water as described before.

The effect of exogenous nitrogen sources on germination was tested by applying KNO_3_ (10 mM), NH_4_NO_3_ (10 mM) or NH_4_Cl (10 mM) to seeds that had been stored in dry conditions for 14 d since seed harvest.

Germination in the presence of the nitrate inhibitor was performed on seeds that had been stored in dry conditions for 28 d since seed harvest. As an inhibitor, 1 mM Na_2_WO_4_ (sodium tungstate, Sigma 223336) was used. Germination was performed with and without 10 mM KNO_3_.

### Nitrate determinations

Nitrate measurements were performed as described in He et al. (2014). In brief, 5 mg of seeds were boiled at 100°C for 15 min in 0.5 ml of 0.5 M HCl and 50 mg l^–1^*trans*-aconitate (internal standard). After centrifuging for 2 min at 13,000 rpm, 200 μl of the supernatant was transferred to an HPLC vial.

HPLC analysis was performed on a Dionex ICS2500 system with an AS11-HC column and an AG11-HC guard column, and eluted with NaOH. The elution procedure was: 0–15 min linear gradient of 25–100 mM NaOH, then 15–20 min of 500 mM NaOH followed by 20–35 min of 5 mM NaOH. Flow rates were 1 ml min^–1^ throughout the run. Contaminating anions in the eluents were removed using an anion trap column (ATC), installed between the pump and the sample injection valve. Anions were determined by conductivity detection. Background conductivity was decreased using an ASRS suppressor, with water as a counterflow. Peaks were identified and quantified using known external standards. The external standard of nitrate was NaNO_3_.

### Ammonium measurement

Ammonium was measured by segmented flow analysis. A 50 mg aliquot of freshly harvested seeds was ground and 5 ml of demi water was added and heated to 80°C for 5 min. Clear supernatant was taken to a new tube. The colorimetric analysis was done on a Skalar San continuous flow analyzer connected to a 1050 autosampler using chemical method 155–324. (Skalar Inc., Buford, GA, USA).

### Gene expression analysis of AtAAH and other genes in the purine pathway

RNA was isolated using the Nucleospin RNA plant kit (Macherey-Nagel: 740949) according to the manufacturer’s protocol with minor modifications as described in Yazdanpanah et al. (2019): 3–5 mg of freshly harvested seeds were used for the extraction. Lysis was performed using 315 μl of buffer RAP, 35 μl of Plant RNA Isolation Aid (Ambion: AM9690) and 3.5 μl of β-mercaptoethanol (Sigma: M6250). Final RNA was eluted in 40 μl of RNase-free water. Quality and concentrations were measured by loading 2 μl of RNA on an Xpose slide 40 (Bioke: TR230300) and measured on an Xpose (Bioke: TR112003). RNA integrity was checked on a 1% agarose gel. cDNA was synthesized from 750 ng of RNA using the iScript cDNA Synthesis Kit (Bio-Rad: 1708890) according to the manufacturer’s protocol. cDNA was diluted 10 times with sterile milliQ water. For each sample 2.5 μl of cDNA, 5 μl of iQ SYBR green supermix (Bio-Rad: 1725125) and 0.5 μl of primer mix (10 μl of work solution) were added and supplemented with water to 10 μl. RT–qPCR was performed on a CFX connect (Bio-Rad).

Sequences of the primers of the target genes are presented in **Supplementary Table S2**. Expression of the genes was normalized based on the expression of two reference genes that are stably expressed in dry seeds: At4g12590 and At4g34270 (Dekkers et al. 2012), as explained by Yazdanpanah et al. (2019). Expression was calculated by using qbasePLUS (Hellemans et al. 2007), which is commercially available software (Biogazelle, Ghent, Belgium, www.biogazelle.com).

### Metabolite extraction and derivatization methods

The metabolite extraction was performed on 24 h imbibed dormant seeds of Col-0 and the *Ataah* mutant based on a previously described method (Roessner et al. 2000), with some modifications. For each genotype, metabolite extractions were performed on four biological replicates. For each sample, 5 mg of seeds pre-cooled in liquid nitrogen were homogenized in 2 ml tubes with two iron balls (2.5 mm) using a micro dismembrator (Mo Bio Laboratory). Then 233 μl of methanol/chloroform (4:3) was added, together with 50 μl of standard (0.13 mg ml^–1^ ribitol), and mixed thoroughly. After 10 min of sonication, 66 μl of MQ water was added to the mixture followed by vortexing and centrifugation (5 min, 15,000 rpm). The methanol phase was collected in a glass vial. Then 166 μl of methanol/chloroform (1:1) was added to the remaining organic phase and kept on ice for 10 min. A 66 μl aliquot of MQ water was added followed by vortexing and centrifugation (5 min, 15,000 rpm). Again the methanol phase was collected and mixed with the previously collected phase. A 60 μl aliquot was dried overnight using a speedvac (room temperature, Savant SPD121). Dried samples were derivatized online as described by Lisec et al. (2006) using a Combi PAL autosampler (CTC Analytics). The derivatized samples were analyzed by a GC-TOF-MS system consisting of an Optic 3 high-performance injector (ATAS) and and Agilent 6890 gas chromatograph (Agilent Technologies) coupled to a Pegasus III time-of-flight (TOF) mass spectrometer (Leco Instruments). A 2 μl aliquot of each sample was introduced to the injector. The details of the GC-TOF-MS method were as described by Carreno-Quintero et al. (2012) with some minor modifications. Detector voltage was set at 1,650 V.

### ABA extraction and detection method

To measure ABA content, 10 mg of frozen dry seeds were ground in a 2 ml Eppendof tube using stainless steel beads. ABA was extracted and purified according to a protocol described by Zhou et al. (2003). ABA content was measured by injecting 10 µl of extract into a Waters Xevo tandem quadruple mass spectrometer equipped with an electrospray ionization source and coupled to an Acquity UPLC BEH C18 column (100 mm) at 0.2 ml min^–1^ with acetonitrile (ACN)/0.1% formic acid (FA), MQ/0.1% FA flow. ABA was quantified using a calibration curve with known amounts of ABA based on the ratio of the summed area of the multiple reaction monitoring transitions for ABA to those for [_2_H_6_]ABA. Data acquisition was performed using MassLynx 4.1 software (Waters, USA).

## Supplementary Material

pcac103_Supp

## Data Availability

No new data were generated or analyzed in support of this research.
