# Supplementary material for: A Role for Allantoate Amidohydrolase (AtAAH) in the Germination of Arabidopsis thaliana Seeds
Source: Plant Cell Physiol. 2022 Jul 21;63(9):1298–308. doi: 10.1093/pcp/pcac103 (PMC9474941; doi:10.1093/pcp/pcac103)
Supplement: pcac103_Supp [file pcac103_supp.zip › pcp-2022-e-00101-File008.docx]

**Supplemental figures**

**

**

**Figure S1**. Expression of *AtAAH* among various plant tissues and developmental periods. Note the strong expression in mature pollen as viewed with the Arabidopsis eFP Browser.

**
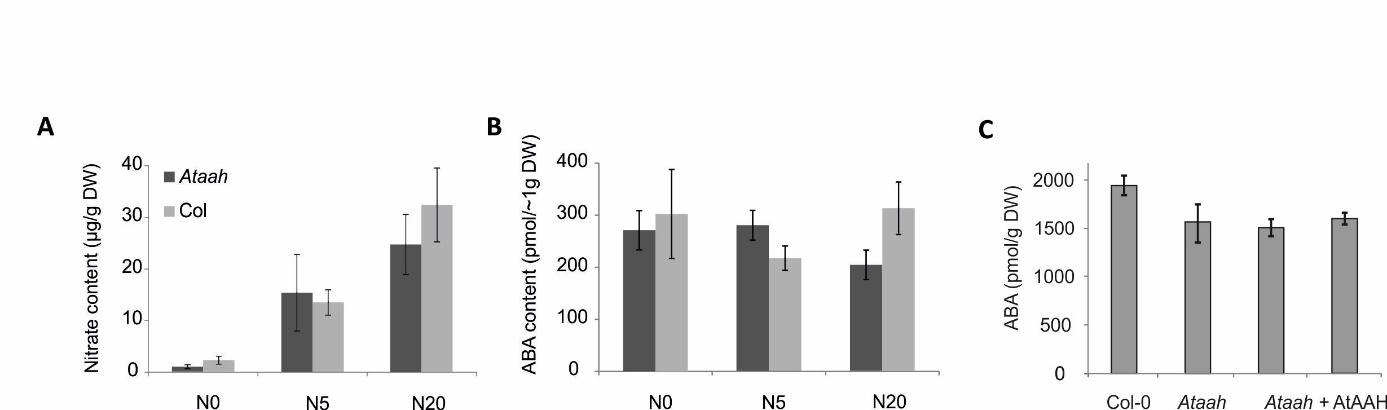
**

**Figure S2.** Nitrate and ABA content of seeds produced under different nitrate regimes. A) Nitrate content (mg/l) and B) ABA concentration (pmol/g DW) of freshly harvested seeds of allantoate amidohydrolase mutant (*Ataah*) and Col-0 after seed maturation on plants exposed to different nitrate regimes (N0, N5 and N20). C) ABA concentration in the *Ataah* mutant and two independent complementation lines (*Ataah* + AtAAH). DW, Dry weight.
