# Supplementary material for: A Role for Allantoate Amidohydrolase (AtAAH) in the Germination of Arabidopsis thaliana Seeds
Source: Plant Cell Physiol. 2022 Jul 21;63(9):1298–308. doi: 10.1093/pcp/pcac103 (PMC9474941; doi:10.1093/pcp/pcac103)
Supplement: pcac103_Supp [file pcac103_supp.zip › pcp-2022-e-00101-File009.docx]

**Supplemental tables**

**Table S1**. Element concentrations in the standard nutrient solution

|  | Macro-element (mM) | | | | | | | | Micro-element (µM) | | | | | |
| --- | --- | --- | --- | --- | --- | --- | --- | --- | --- | --- | --- | --- | --- | --- |
| element | N | K | Ca | Na | Mg | Cl | S | P | Fe | Mn | Zn | B | Cu | Mo |
| concentration | 5 | 2.9 | 2 | 0.4 | 0.5 | 0.4 | 1.1 | 0.5 | 3.5 | 2.6 | 3.0 | 20 | 6.6 | 0.2 |

The pH of the solution was 7.0

**Table S2**. Primer pairs used for qrtPCR analyses

| **Gene** | **Primer sequence (5’-3’)** | |
| --- | --- | --- |
| **AtAAH/AT4G20070** | F: CATTGAACGAAAGCACGATG | R: TCCTGCTCCACTCATTAGCA |
| **AtALN/AT4G04955** | F: AGCAAAAGGCAAAGGAGACA | R: CTGGAATCTCTTCGGCTGAG |
| **UGLYAH/AT4G17050** | F: ATCGCTTGGGTGATAACTGG | R: CAGCATACCATTGAGGGACA |
| **URE/AT1G67550** | F: GCATTTCGTGGGAGAACAAT | R: TGGTTGATGACGGGAGTACA |
| **UAH/AT5G43600** | F:  ATCTGCCTGCTGTAGCCACT | R: AAACACCGACAACACCATCA |
